# A Fast Multi-Scale Generative Adversarial Network for Image Compressed Sensing

**DOI:** 10.3390/e24060775

**Published:** 2022-05-31

**Authors:** Wenzong Li, Aichun Zhu, Yonggang Xu, Hongsheng Yin, Gang Hua

**Affiliations:** 1 School of Information and Control Engineering, China University of Mining and Technology, Xuzhou 221008, China; wenzongli@cumt.edu.cn (W.L.); xygang@cumt.edu.cn (Y.X.); yhs@cumt.edu.cn (H.Y.); 2 School of Computer Science and Technology, Nanjing Tech University, Nanjing 211800, China; aichun.zhu@njtech.edu.cn

**Keywords:** compressed sensing, generative adversarial network, lightweight multi-scale residual block, multi-scale sampling

## Abstract

Recently, deep neural network-based image compressed sensing methods have achieved impressive success in reconstruction quality. However, these methods (1) have limitations in sampling pattern and (2) usually have the disadvantage of high computational complexity. To this end, a fast multi-scale generative adversarial network (FMSGAN) is implemented in this paper. Specifically, (1) an effective multi-scale sampling structure is proposed. It contains four different kernels with varying sizes so that decompose, and sample images effectively, which is capable of capturing different levels of spatial features at multiple scales. (2) An efficient lightweight multi-scale residual structure for deep image reconstruction is proposed to balance receptive field size and computational complexity. The key idea is to apply smaller convolution kernel sizes in the multi-scale residual structure to reduce the number of operations while maintaining the receptive field. Meanwhile, the channel attention structure is employed for enriching useful information. Moreover, perceptual loss is combined with MSE loss and adversarial loss as the optimization function to recover a finer image. Numerous experiments show that our FMSGAN achieves state-of-the-art image reconstruction quality with low computational complexity.

## 1. Introduction

Compressed sensing (CS) is an emerging information acquisition technique, which overcomes the Nyquist–Shannon acquisition theorem’s limitations and implements signal sampling and compressing simultaneously [1]. The theory implies that when a signal $x\in R^{n}$ is compressible or sparse in a certain domain Ψ, it can compressed and measured by the measurement matrix Φ, and inferred accurately from y=Φx, where $\Phi \in R^{m\times n}$ with m≪n**.** The m/n is defined as the sampling rate. Due to the captivating sampling performance of CS, it is attractive for numerous applications, including video CS [2], single-pixel camera [3], snapshot compressed imaging [4] and magnetic resonance imaging [5].

The study of CS mainly focuses on the sampling pattern and recovery approaches at present. In terms of sampling, lots of approaches [6,7,8,9] have been developed and most of them perform well. Measuring images in the multi-layer transform domain is dubbed multi-scale sampling, whereas measuring images in the original domain is dubbed single-scale sampling. With the intelligent utility of prior knowledge (structure, statistical dependencies, etc.), multi-scale sampling achieves better reconstruction quality than single-scale sampling but has received less attention [6,7]. Most scholars focus on single-scale sampling and have designed various measurement matrices [8,9]. Usually the well-designed or learned single-scale measurement matrix can acquire well-accepted reconstruction quality. However, these methods [8,9] suffer from aliasing artifacts for more attention to low-frequency information. Additionally, measuring and reconstruction are usually implemented separately, thus their performance is limited.

**Figure 1:**
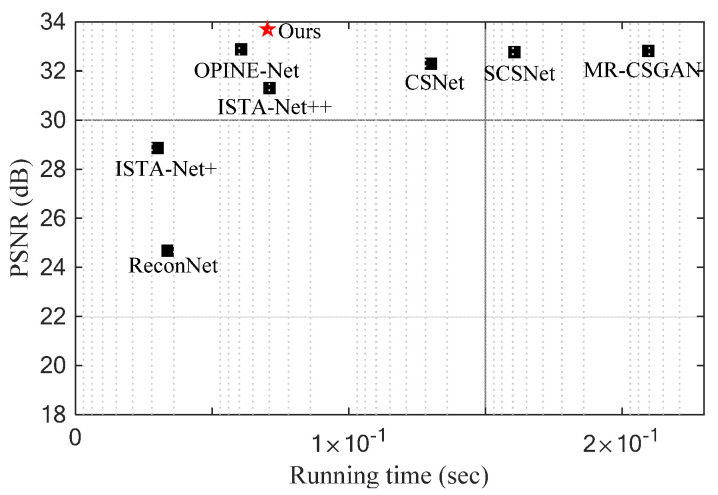
Running time and recovery quality comparison. The running time is the average time for recovering an image in the Set5 dataset. The recovery quality is the average PSNR of the image in the Set5 dataset under a sampling rate of 0.1.

The recovery of CS is treated as an inverse problem. For this, some classical algorithms have been proposed, including greedy algorithms [10,11], convex optimization algorithms [12,13] and iterative thresholding algorithms [14]. Greedy algorithms are easily affected by the local optimal solution, so recovery quality is limited. Convex algorithms and iterative thresholding algorithms usually implement multiple iterations for better recovery quality and are thus more time consuming. Therefore, while many works have been devoted to designing a fast method, reconstruction quality is lost [15,16]. Recently, deep neural networks have shown super performance in a variety of image processing tasks [17,18,19]. Some representative network structures, including convolutional neural networks (CNN) and generative adversarial networks (GAN) are also employed to image CS reconstruction. With the powerful learning ability of deep learning, these data-driven neural network models for image CS (DICS) have impressive reconstruction quality by directly learning the mapping from the compressed measurements to the raw image. We also notice that due to the alternating training of generator and discriminator, the image reconstructed by the method based on GAN is more authentic than that based on CNN [20]. DICS is obviously superior to classical methods in image recovery quality and speed. However, similar to the evolution of classical methods, recent DICS often exchange more time resources for less improvement in image reconstruction quality, as shown in Figure 1. This is mainly because DICS often stacks numerous of the same blocks to obtain high-resolution images and each block cannot help recover images effectively. For example, in [21], the author proposes a serial structure based on CNN. Because the structure is relatively simple, the quality of image reconstruction can be further improved. In [20], the author develops a multi-scale residual block. The block can capture multi-scale image features, but it needs more time to process images and lacks the fusion of each channel feature. Therefore, there is an urgent need for efficient DICS to promote the application of image CS in high real-time scenes.

To solve the above problems, a fast multi-scale generative adversarial network (FMSGAN) is proposed. Specifically, there are two improvements in the FMSGAN: (1) inspired by [12], we propose a novel multi-scale sampling structure (MSS), which involves four convolution layers with different kernel sizes and a concatenated layer. The former three parallel convolution layers decompose images at each scale independently to obtain features with multiple resolutions. The later convolution layer is applied for sampling concatenated features. Our MSS can capture different levels of spatial features at multiple scales and help improve reconstruction quality. (2) We propose a lightweight multi-scale residual block (LMSRB), in which only the 3 × 3 convolution layer and the concatenated layer are used. There are three bypasses in the LMSRB and the corresponding structures: one 3 × 3 convolution layer, two serial 3 × 3 convolution layers and three serial 3 × 3 convolution layers, respectively. The serial convolution layers with a small kernel size have the same receptive field as a convolution layer with a large kernel size. So images of features at different scales can be learned by the LMSRB, thus enriching feature representation. Furthermore, a channel attention structure is applied to give different weights for every LMSRB output feature map to better enhance useful information. Because of the LMSRB and the channel attention structure, the FMSGAN is capable of high-resolution images and low computational complexity. Additionally, we introduce perceptual loss to refine the loss function. To verify the performance of our FMSGAN, we perform extensive experiments on three datasets, and the results show the merits of our model.

The contributions are summarized as follows:

(1) A fast multi-scale generative adversarial network is proposed for image CS. The generator and discriminator are alternate training to ensure the reconstructed images are more realistic.
(2) A multi-scale sampling structure is proposed, which improves image reconstruction quality through joint training with the reconstruction network.
(3) A novel lightweight multi-scale residual block (LMSRB) is proposed, which is combined with the channel attention structure to better tradeoff between reconstruction performance and efficiency. Due to the high efficiency of the LMSRB, the image is reconstructed at high speed.
(4) Our FMSGAN achieves state-of-the-art performance on three datasets.

## 2. Related Work

Recently, compressed sensing has became a fascinating research area. It has a wide range of applications, especially in wireless sensor networks (WSN) and internet of things (IoT). In [22], a compressed sensing-based scheduling scheme was developed to conserve energy in WSN and IoT. The scheme firstly addresses the question of “how many sensor nodes should be activated to sense and transmit”, then forces each sensor node to transmit only m≪n measurements to its next-hop node, for extraordinary performance in energy conservation. In [23], a compressed sensing framework is proposed for WSN and IoT. The authors demonstrate that the framework can be utilized to recover the compressible information data into a variety of information systems and will contribute to saving energy and communication resources. For reconstructing a diffusion field from spatiotemporal measurements, Mohammad et al. [24] exploit the intrinsic property of diffusive fields as side information and propose a diffusive compressed sensing method, which produces estimates of higher accuracy than that of classic CS. In [25], the authors consider power-hungry sensors, introduce compressed sensing and distributed compressed sensing to WSN and provide great energy efficiency. Hoover et al. [26] merge the CS process with existing methods of collecting spectral images and expand the stacked-color image sensor to use more colors or a wider range of wavelengths, which obtain a higher spectral resolution. There are more image CS works on the sampling pattern and recovery method. In the sampling process, researchers find that multi-scale sampling can extract different levels of image feature information [7,27]. By enriching the multi-level contents of the model, multi-scale sampling can enhance both sampling quality and recovery quality. As a simple implementation of multi-scale sampling, radial Fourier subsampling [28] is usually applied in bioimaging for its conversion characteristics between spatial and frequency domains but is not verified by more images. Flowers first decomposes images in the wavelet domain, then implements adaptive sampling of each wavelet sub-band independently and finally smooths the measurements to effectively obtain multi-scale information [6]. The W-DCS [27] applies wavelet transform for multi-scale compressed sensing. It is able to extract the measurements in multiple decomposed scales. For Kronecker CS, a multi-scale sampling method is developed, which achieves high reconstruction quality and high computational complexity [7]. Despite these wavelet-based methods [6,7,27] improving image reconstruction quality, they require that the input image size meet the integer multiple of 2. More cases of multi-scale sampling are in [29,30,31]. In LAPRAN [29], a series of measurements at different resolutions are defined for a given sampling rate. Each group of measurements is fed into the corresponding reconstruction stage, thus multi-scale sampling is implemented. However, a heuristic measurement assignment is commanded for each rate. As a scalable network, SCSNet [30] creates multiple levels of reconstruction quality through a variety of stages of reconstruction. Its primary reconstruction module supports more low-frequency contents. However, SCSNet prefers to solve the adaptation sub-rate issue rather than devise a multi-scale sampling method. In MS-CSNet [31], a series of measurements are defined. The authors train the network with the obtained measurements corresponding to the smaller sub-rate and reuse them at the larger sub-rate, in which the low-frequency information is shared in the high-level recovery stage. However, MS-CSNet does not display the subjective reconstruction of images. Therefore, various rigorous studies on multi-scale sampling are required.

In the recovery process, image CS infers the raw image from given measurements. For this, conventional CS approaches [10,32,33,34] mainly depend on sparsity priors to iteratively optimize the sparsity-regularized problem. Examples of such approaches include orthogonal matching pursuit (OMP) [10], basis pursuit (BP) [32], the iterative shrinkage thresholding algorithm (ISTA) [33] and the alternating direction method of multipliers (ADMM) [34]. To further enhance recovery performance, researchers established more detailed structures based on wavelet tree sparsity [35], non-local information [36], minimal total variation [37] and simple representations in adaptive bases [38]. However, these conventional CS approaches are usually afflicted with high computational complexity caused by hundreds of iterations.

Deep unfolding approaches usually integrate the deep networks with the iterative optimizers for image reconstruction. Metzler et al. [39] were the first to propose a learned DIT (LDIT), which combines the iterative DIT algorithm with a denoising CNN. Zhang et al. implement a set of deep unfolded versions of the ISTA algorithm, named ISTA-Net+ [9], OPINE-Net [40] and ISTA-Net++ [41], respectively. The difference is that ISTA-Net applies random measurement and recovery of the image block by block, the OPINE-Net designs a learning matrix and trains it jointly with the whole network and the ISTA-Net++ achieves multi-rate sampling and recovery in one model by a dynamic unfolding method. Moreover, based on the AMP algorithm, Zhang et al. [42] propose the AMP-Net to recover images with high quality and speed. The main limitation of such unfolding approaches is that they usually have the disadvantage of poor image recovery quality under a low sampling rate due to adopting a plain network structure.

Deep straightforward approaches can directly learn the mapping between measurements and original images free from any constraints. Mousavi et al. [43] were the first to adopt a stacked denoising autoencoder (SDA) for image reconstruction while the applied fully connected network (FCN) results in numerous parameters. ReconNet [44] is the first approach to reconstructing the image from measurements via CNN, which has better recovery quality and fewer parameters. Subsequently, several CNN-based recovery approaches [21,45] are proposed. In MR-CSGAN [20], the authors adopt the generative adversarial network to recover images, whose generator and discriminator were alternately trained, so that the recovered image is more realistic. Recently, a novel block-based image CS network (BCSnet) [46] was proposed. By exploiting image intercorrelation, BCSnet achieves impressive performance. However, deep straightforward approaches often acquire limited performance improvement with many computational resources and are thus not suitable for high real-time applications.

## 3. Methods

**Figure 2:**
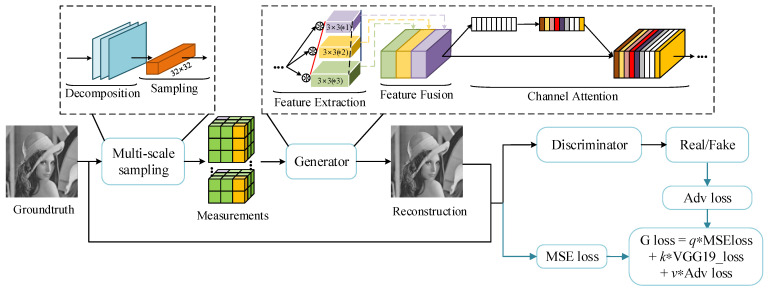
The overall architecture of the proposed FMSGAN.

In this part, we display the overall architecture of the FMSGAN, as shown in Figure 2. The raw image is sampled by the multi-scale sampling structure, and recovered by the generator, respectively. Both the raw image and the corresponding recovered image will be fed into the discriminator, in which the recovered image is distinguished from the raw image.

### 3.1. Multi-Scale Sampling Structure

**Figure 3:**
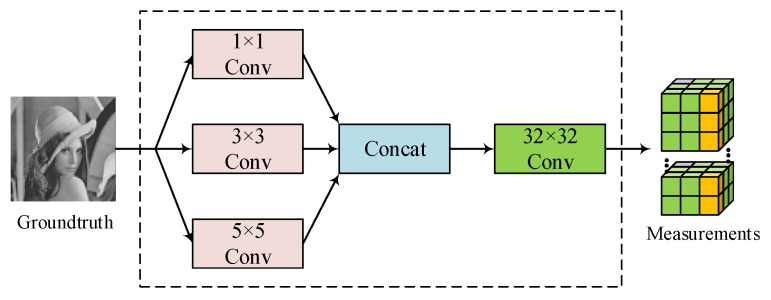
The structure of multi-scale sampling.

In the multi-scale sampling structure, the raw image is divided into multiple non-overlapping blocks of size ${l\;\times \;B}_{1}{\;\times \;B}_{2}$, where *l* denotes the image channels. To obtain measurements, a set of convolutions are utilized to realize the multi-scale decomposition and sampling of the image block. The first-level decomposition can be formulated as:

(1) $$x_{l_{1}}^{1}{=W}_{l_{1}}^{1}{*x}^{0}$$

(2) $$x^{1}=\left[x_{1}^{1},x_{2}^{1},\ldots ,x_{c_{1}}^{1}\right]$$

where ∗ is the convolution operation, $W_{l_{1}}^{1}$ denotes different convolution kernels in the first-level decomposition, $l_{1}\in {1,2,\ldots ,c}_{1}$ is the identifier of convolution kernels, $x^{0}$ denotes the image block with a size of ${l\;\times \;B}_{1}{\;\times \;B}_{2}$ and $x^{1}$ denotes the output of the first-level decomposition. If the image is decomposed *n* times, the measurements are expressed as:

(3) $$x^{n}{=W}_{l_{n}}^{n}{*x}^{n-1}{=W}_{l_{n}}^{n}{*W}_{l_{n-1}}^{n-1}*\cdot \cdot \cdot {*(W}_{l_{1}}^{1}{*x}^{0})$$

where $x^{n}\in R^{l_{n}{\times m\times b}_{1}{\times b}_{2}}$, $l_{n}$ is the number of convolution kernels at $n_{\mathrm{th}}$-level decomposition, *m* is the number of output channels of every convolution and $b_{1}{\times b}_{2}$ denotes the size of output features. For a given sampling rate *r*, there is $l_{n}{\;\times \;m\;\times \;b}_{1}{\;\times \;b}_{2}{=r\;\times \;l\;\times \;B}_{1\;}{\times \;B}_{2}$. The multi-scale sampling structure is shown in Figure 3. Firstly, three parallel convolutions—1 × 1, 3 × 3 and 5 × 5—are employed to decomposition image and output features. Convolution kernels with different sizes have different receptive fields, so different levels of feature information can be obtained. Then, the features are synthesized by the concatenated layer. Finally, a convolution layer with kernel size 32 × 32 and step size 32 × 32 is applied to output the measurements. Specially, all convolutions are no bias and activation. In experiment, n is set to 2 for fast sampling. Both $B_{1}$ and $B_{1}$ are set to 64 in the training phase. The test image is not forced to be segmented, as long as the size $N_{1}{\;\times \;N}_{2}$ meets $N_{1}{\;\times \;N}_{2}{=32k}_{1}{\;\times \;32k}_{2}$, where $k_{1}$ and $k_{2}$ are positive integers. Otherwise, image overlapping segmentation or image filling will be applied.

### 3.2. Generator Structure

**Figure 4:**
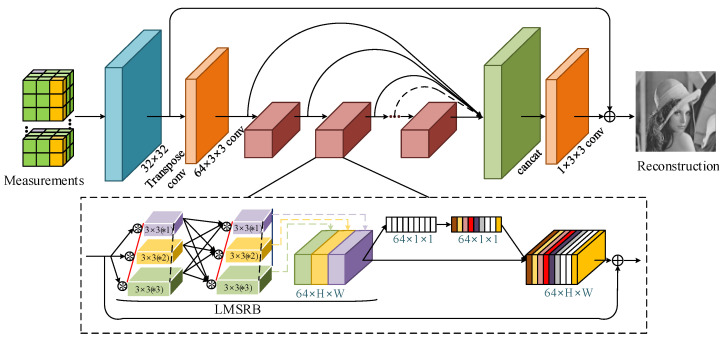
The overall architecture of the generator.

The generator can transform the measurements into a high-resolution image, which involves two processes: initial recovery and deep recovery. The architecture of the generator is shown in Figure 4. The initial recovery uses a deconvolution layer with kernel size 32 × 32 to recover images from the corresponding measurements. In the deep recovery process, we firstly apply a convolution with 64 channels to increase the number of feature maps. Then, nine LMSRBs combined with channel attention modules are adopted to deep recovered images in a single connection. The structure of the LMSRB is shown in the scribed part in Figure 4. The input features are processed by the LMSRB, in which multiple information at different bypasses is shared to capture image features at multiple scales. There are two of the same pyramid-like convolution structures in the LMSRB and each structure contains three parallel convolution groups, corresponding to one 3 × 3 convolution, two serial 3 × 3 convolutions and three serial 3 × 3 convolutions, respectively. The pyramid-like convolution can provide multi-scale feature representation and the serial 3 × 3 convolutions are able to decrease the number of operations while maintaining the receptive field. At the same time, the channel attention model is employed to acquire the contribution of each LMSRB output channel through learning and assigning different weight coefficients to each channel, so as to strengthen the important features. Moreover, the residual connection is used for the stability of network training. Subsequently, a concatenated layer connected to every channel attention model is adopted to enrich feature representation. A convolution layer with 3 × 3 is employed to decrease the number of feature maps and output the deep recovered images. Finally, the initial recovered image and the deep recovered image are added to acquire the reconstructed image.

### 3.3. Discriminator Structure

**Figure 5:**
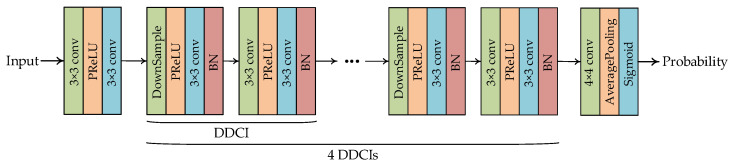
The structure of the discriminator.

The design of the discriminator refers to [20], which contains convolution layers, batch normalization layers, Leaky Relu functions and sigmoid function, as shown in Figure 5. In particular, the convolution layer is added behind each batch normalization layer to enhance the discrimination ability of the discriminator by increasing the weight parameters. Note that there are some similar operations in the identification process. For simplicity, the single operation of dimension decrease and channel increase for the feature map is named DDCI. The recovered image and the corresponding original image generated by the generator is fed into the discriminator and then the probability of sample classification is obtained.

### 3.4. Cost Function

Inspired by [47], the MSE loss, perceptual loss, and adversarial loss are combined as the cost function of our FMSGAN. The MSE loss often converges quickly but it is hard to reconstruct some lost uncertain high-frequency details, leading to poor visual quality. Recently, perceptual loss has outperformed MES loss in some computer vision tasks. It is capable of preserving structure and details, so was introduced into our model. The pixel-level MSE loss is formulated as:

(4) $${\;l}_{\mathrm{MSE}}=\frac{1}{\mathrm{HV}}\overset{H}{\underset{i=1}{\sum }}\overset{V}{\underset{j=1}{\sum }}{{\left(G(I\right)}_{i,j}{-I}_{i,j})}^{2}$$

where G(⋅) represents the generator, ${G(I)}_{i,j}$ denotes the image created by the generator, $I_{i,j}$ is the input image, and H and V represent the number of pixels in the horizontal and vertical directions of the input image, respectively. The VGG19 loss is implemented for obtaining high-level perceptual information, which is expressed as:

(5) $$l_{\mathrm{VGG}19}=\frac{1}{H_{x,y}V_{x,y}}\overset{H_{x,y}}{\underset{i=1}{\sum }}\overset{V_{x,y}}{\underset{j=1}{\sum }}{\left(\phi_{x,y}{\left(G\left(I\right)\right)}_{x,y}{-I}_{x,y}\right)}^{2}$$

where $\phi_{x,y}(\cdot )$ represents the feature map captured by the *j*th convolution layer before the *i*th max-pooling layer in the VGG19 network. $H_{x,y}$ and $V_{x,y}$ denote the size of the respective feature maps in the VGG19 network. Here, the $\phi_{x=5,\;y=4}$ of the VGG19 network is chosen as the final output layer for the feature map. Through minimizing adversarial loss to optimize the parameters, more indistinguishable images created by the generator are applied to trick the discriminator, which also promotes the performance of the discriminator. The adversarial loss is as follows:

(6) $$l_{\mathrm{Adv}}=\overset{M}{\underset{m=1}{\sum }}1-D(G(I))$$

where D(⋅) represents the discriminator, *D*(*G*(I)) denotes the probability that the recovered image *G*(I) is real and M represents the batch size during each training iteration. The final cost function is defined as:

(7) $$l_{\mathrm{total}}=q{*l}_{\mathrm{MSE}}{\;+\;k*l}_{\mathrm{VGG}19}{+v*l}_{\mathrm{Adv}}$$

## 4. Experiments

In this section, we first conduct a comparison with some state-of-the-art approaches to verify the performance of the proposed model. Then, the effectiveness of the MSS and the LMSRB are verified by ablation experiments. The discussion and interpretation of the experimental results are also provided.

### 4.1. Datasets

All experiments are adopted on five datasets: DIV2K [20], Set5 [45], Set11 [42], Set14 and BSDS100 [21]. DIV2K is a high-resolution dataset, which contains 800 color images and is our training dataset. Random clipping, translation and rotation are utilized to expand the training data. In particular, all images in DIV2K are cropped into sub-images with a size of 64 × 64. Set11 is employed to validate. Additionally, we use Set5, Set14 and BSDS100 as the test datasets.

### 4.2. Implementation Details

All experiments are performed using PyTorch 1.6 platform with 1 GeForce RTX1080Ti GPU. The Adam is used as the generator’s optimizer and the initial learning rate is set to 0.0004. After every 180 iterations, the learning rate will be divided by 2. The SGD is used as the discriminator’s optimizer and the learning rate is set to 0.0004. Assigning different optimizer and learning rates, updating strategies for the generator and discriminator, is beneficial for the stable training of the model. We use four sampling rates to sample images—1%, 4%, 10% and 25%—and choose 10, 41, 102 and 256 as the numbers of corresponding measure convolution output channels. We choose the structural similarity index (SSIM) and peak signal-to-noise ratio (PSNR) as the evaluation index for recovery quality.

### 4.3. Results

#### 4.3.1. Comparison to Other State-of-the-Art Methods

**Figure 6:**
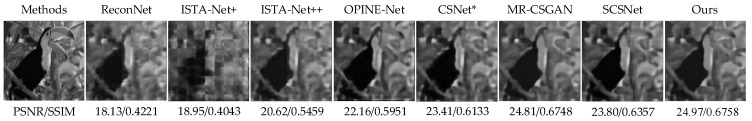
Comparison of visual recovery on bird from Set5 at a sampling rate of 1%.

**Figure 7:**
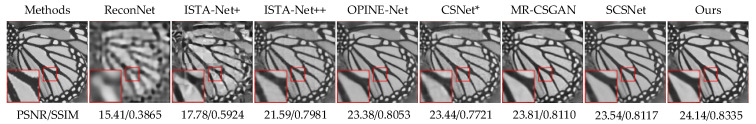
Comparison of visual recovery on butterfly from Set5 at a sampling rate of 4%.

**Figure 8:**
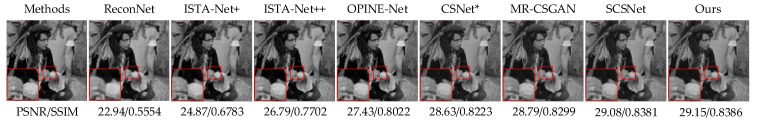
Comparison of visual recovery on man from Set14 at a sampling rate of 10%.

**Figure 9:**
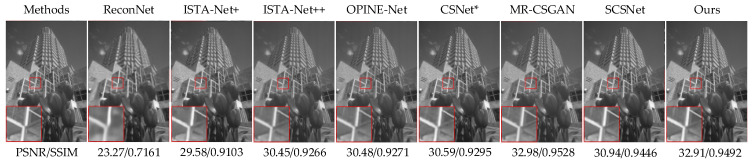
Comparison of visual recovery on building from BSDS100 at a sampling rate of 25%.

We compare our FMSGAN with some state-of-the-art methods, i.e., ReconNet [44], ISTA-Net+ [9], SCSNet [30], CSNet* [21], OPINE-Net [40], ISTA-Net++ [41], AMP-Net [42] and MR-CSGAN [20], on three datasets, namely Set5, Set14 and BSDS100, to verify its recovery quality and running speed. The recovery quality comparisons are shown in Table 1, Table 2 and Table 3 and running time comparisons are shown in Table 4. In particular, we introduce the mean and standard deviation (SD) to compare reconstruction times in a statistical manner. PSNR and SSIM results show that our FMSGAN performs better. On the Set5 dataset, the FMSGAN almost achieves the highest PSNR and SSIM results. Specifically, at the four sampling rates, i.e., 1%, 4%, 10% and 25%, the proposed model achieves 0.15, 0.46, 0.85 and 0.36 dB and 0.0245, 0.0229, 0.0147 and 0.0029 gains in PSNR and SSIM compared with MR-CSGAN. The improvement in reconstruction quality is mainly due to prior knowledge captured by the multi-scale sampling structure. On the Set14 dataset, the proposed model achieves average 6.72, 3.96, 0.59, 0.76, 1.03, 2.11, 0.31 and 0.16 dB and 0.2290, 0.1202, 0.0227, 0.0305, 0.0172, 0.0557, 0.0144 and 0.0190 gains in PSNR and SSIM compared with the other eight methods, as shown in Table 2. Compared with ReconNet, our model achieves 5.10, 6.10, 7.08 and 8.60 dB and 0.1882, 0.2550, 0.2551 and 0.2175 gains in PSNR and SSIM at the four sampling rates. On the BSDS100 dataset, the proposed model achieves average 5.78, 3.81, 0.38, 0.27, 1.56, 2.33 and 0.20 dB and 0.1990, 0.1246, 0.0134, 0.0227, 0.0312, 0.0625 and 0.0009 gains in PSNR and SSIM compared with the other seven methods, as shown in Table 3. Compared with OPINE-Net, our model achieves 2.06, 1.52, 1.37, and 1.28 dB and 0.0527, 0.0337, 0.0242 and 0.0143 gains in PSNR and SSIM at four sampling rates. We find that the AMP-Net has a higher PSNR in image recovery at a sampling rate of 25%, which indicates that the performance of the FMSGAN needs to be further improved. We also notice that our FMSGAN and the suboptimal method MR-CSGAN demonstrate similar reconstruction quality on the BSDS100 dataset. This is because BSDS100 is a high-resolution dataset, which needs a more complex affinity for image CS recovery. Due to the application of 3 × 3 convolution, our FMSGAN requires less computation; therefore, its learning ability decreased slightly. We assumed that the effect of recovery quality decreasing slightly is negligible compared to the decrease processing time. Later, we will analyze the computational complexity of the eight methods. For further comparison, we calculate the standard deviation (SD) of PSNR and SSIM of each model at four sampling rates on three datasets, as shown in Table 1, Table 2 and Table 3. Compared with deep straightforward approaches, deep unfolding approaches, i.e., ISTA-Net+, ISTA-Net++, OPINE-Net and AMP-Net, achieve higher values in both PSNR SD and SSIM SD. With high SD, one model can have a rich ability to deal with the measurements corresponding to different sampling rates. Benefiting from iterative thresholding algorithms, deep unfolding approaches usually have outstanding performance. PSNR SD and SSIM SD of our model on three datasetsare 4.9791, 3.9615, 3.1427 and 0.1144, 0.1313, 0.1340, respectively and are among the highest in deep straightforward approaches. This means that our model can maintain better recovery performance at a low sampling rate while achieving high SD, which remedies the deficiency of deep straightforward approaches. Subjective reconstruction comparisons are shown in Figure 6, Figure 7, Figure 8 and Figure 9, from which can find that, compared with other methods, the FMSGAN is better able to retain more details and sharper edges.

Table 4 is the reconstruction time comparisons between different CS approaches for recovering a 256 × 256 image in the Set11 dataset at a sampling rate of 10%. We test ISTA-Net+, OPINE-Net, ISTA-Net++ and MR-CSGAN on our platform (1 GeForce RTX1080Ti GPU) with their original codes and the results of SCSNet, ReconNet, and CSNet are supported by [20]. In Table 4, we can see that the time to reconstruct a 256 × 256 image by our FMSGAN is only 0.0406 s, less than that of SCSNet, ISTA-Net++ and MR-CSGAN and nearly 1/3 of that of the MR-CSGAN. The comparison results display that our FMSGAN is capable of fast image CS reconstruction.

#### 4.3.2. Ablation Study

1. The MSS

In this section, we evaluate the performance of the MSS. For a fair comparison, only the last convolution layer in the MSS is kept. Table 5 shows the PSNR comparison between w/MSS and w/o MSS tested on the Set14 dataset at four different sampling rates. It is easy to see that the MSS structure greatly facilitates recovery performance across all sampling rates, with the most obvious improvement up to 0.37 dB, which convincingly demonstrates the effectiveness of the MSS.

2. The LMSRB vs. the MSRB

**Figure 10:**
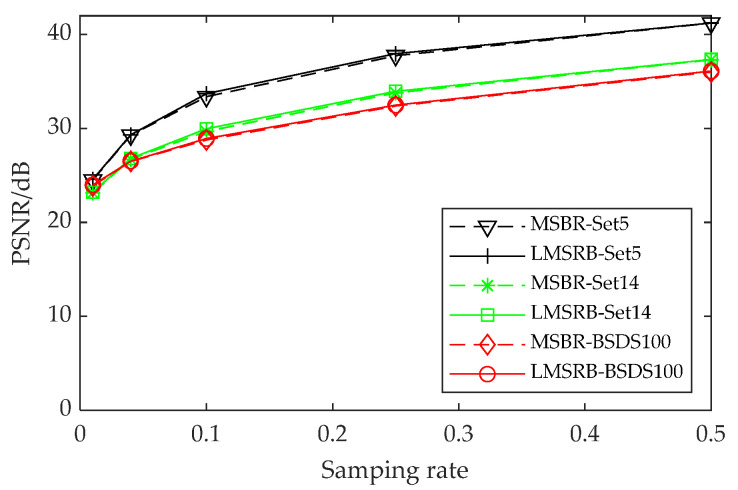
PSNR comparisons of two methods on three datasets at different sampling rates.

To verify the effectiveness of the LMSRB, we replace it with the MSRB [20] in the FMSGAN and carry out experiments. Reconstruction quality comparisons and running speed comparisons are shown in Figure 10 and Table 6, respectively. Figure 10 shows the PSNR of two models tested on the Set5, Set14 and BSDS100 datasets at different sampling rates. We observe that our LMSRB acquires a higher PSNR at sampling rates of 1%, 4%, 10% and 50%, the model with a MSRB has a higher PSNR at a sampling rate of 25% and there is a slight difference between the two models in image recovery quality. Table 6 shows the running time of two models tested on Set11. We find that the time to recover a 256 × 256 image by the FMSGAN is always evidently less than that of the model with a MSRB; this is because the number of feature maps in the LMSRB is the same as that of the MSRB, whereas the number of operations in the LMSRB is significantly less than that of the MSRB. The comparison results show the better performance of the LMSRB.

3. Effect of cost function

**Figure 11:**
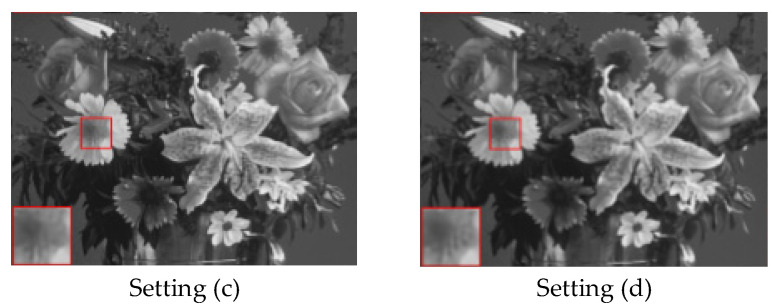
Comparison of visual recovery on flowers from Set14 at a sampling rate of 10%.

For further analysis of the proposed model, various settings of the cost function are concerned and the corresponding recovery performance is shown in Table 7. In particular, we maintain pixel loss as the main part of the cost function. From Table 7, one can clearly observe that setting (d) achieves the best reconstruction performance. Comparing setting (a) and setting (c), we notice that perceptual loss could promote the final recovery results. It seems that adversarial loss has little contribution to recovery performance if only concerning PSNR. Therefore, we display the image subjective reconstruction result in Figure 11. One can see that adversarial loss is capable of supporting better visual results and helps keep context details.

**Figure 12:**
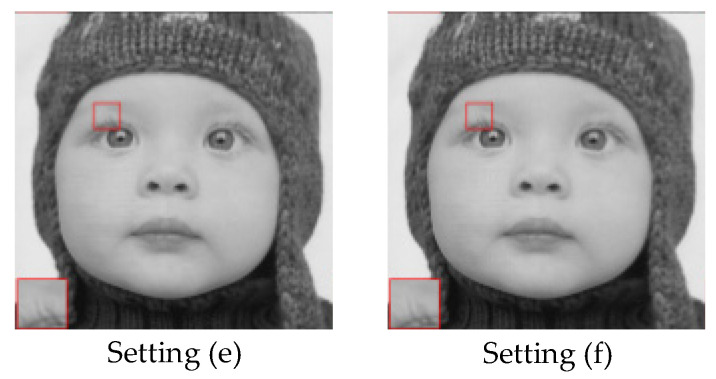
Comparison of visual recovery on baby from Set5 at a sampling rate of 10%.

Furthermore, we also explore the impact of different coefficient combinations of cost function on reconstruction performance, as shown in Table 8. It can be seen that the coefficient of perceptual loss has an obvious influence on the final reconstruction. Whether *k* is greater or less than 0.006, the reconstruction performance will be worse. This means that perceptual loss should be well coordinated with the whole cost function. For adversarial loss, we tend to verify its performance through visual results provided in Figure 12. From Figure 12, we find that the influence of *v* on the final reconstruction is nearly negligible.

### 4.4. Discussion

As far as we know, a lot of DICS methods have been proposed. Most of them are committed to improving reconstruction quality instead of reducing the running time of image reconstruction. We believe that reducing the time complexity of reconstruction is also of great significance, especially in some real-time scenarios, such as automatic driving.

We introduce GAN to implement image CS. From Table 1, Table 2 and Table 3, we can see that the proposed FMSGAN almost achieves the highest PSNR and SSIM values on the three datasets, an exceptional reconstruction effect. This is due to the advantage of multi-scale information. In the FMSGAN, two main structures, a MSS and a LMSRB, are proposed. In the sampling stage, the MSS extracts multi-scale information through convolution kernels of different sizes. Convolution with different kernel sizes has different receptive fields, which can capture more correlation information between pixels. In the recovery stage, the LMSRB extracts and synthesizes multi-scale information through convolution kernels of multiple branches and different depths. After the LMSRB, the image has rich feature representations, but some of them are redundant Therefore, we introduce the channel attention module to filter invalid features and enhance useful features, so as to improve reconstruction quality. We also notice that our FMSGAN achieves a lower PSNR and a higher SSIM compared with AMP-Net at a sampling rate of 25%, which is mainly because the AMP-Net employs the added deblocking model. In the meantime, there is only the mean square error loss that is applied in AMP-Net’s loss function and the mean square error loss tends to optimize pixel-level errors, so the AMP-Net acquires a higher PSNR instead of a balance between PSNR and SSIM. The reconstruction performance of various methods for different datasets is different and most of them achieve the worst reconstruction effect on the BSDS100 dataset. This may be because the BSDS100 dataset is the largest of the three test sets. It contains a wide variety of high-resolution images, which require more complicated mapping during reconstruction. In Table 4, we find that the time to reconstruct a 256 × 256 image by the FMSGAN is only 0.0406 s, less than that by SCSNet, ISTA-Net++ and MR-CSGAN, and is nearly a 1/3 of that by MR-CSGAN. This is mainly because we apply concatenated 3 × 3 convolution instead of large-scale convolution in the LMSRB, which obviously reduces the number of operations. In SCSNet, the author achieves better reconstruction quality through a multi-stage reconstruction strategy, but needs high time complexity. It is necessary to design a more efficient network structure.

GAN itself is prone to the problems of non-convergence and model collapse. In the design of the model, we try to keep the parameters of the discriminator and the generator in the same order of magnitude, and ensure that the parameters of the generator are slightly more than those of the discriminator, which can give full application to the discriminator’s ability without affecting the reconstruction ability of the generator. In our experiment, the number of parameters of the generator are no more than twice that of the discriminator. Further, we assign different optimizers and learning rate update strategies to the generator and discriminator, respectively, so that our model can avoid falling into the problem of mode collapse. For model convergence, we design the cost function based on pixel loss, adversarial loss and perceptual loss. Pixel loss helps the model converge quickly, so we give it a large weight. Adversarial loss and perceptual loss are treated as the auxiliary parts of the cost function, which are assigned small weights. Taking advantage of the design of the function, the model can be trained stably.

In the future, scholars can pay more attention to video compressed sensing. As an ordered image group, video has more redundant information available in the temporal domain and the spatial domain. Making full use of this redundant information will achieve higher-quality data compression, which is of significance.

## 5. Conclusions

In this paper, we present a generative adversarial network-based image compressive model. Specifically, a multi-scale structure is applied for capturing multi-level information to improve reconstruction. An LMSRB structure is applied for deep reconstruction. With the application of multiple 3 × 3 convolutions, multi-scale information of features is better acquired and the number of operations is evidently decreased, which is helpful for capturing detail and recovering images quickly. At the same time, perceptual loss is introduced to enhance the visual quality of the recovered image. Experimental results show that our FMSGAN achieves better reconstruction quality and fast recovery speed against some state-of-the-art methods on three datasets.

Despite the superiority of the FMSGAN, further improvement can still be achieved in the reconstruction of DICS. With further in-depth research on deep learning, some novel networks with brilliant performance can be derived, which are capable of powerful information capture and feature extraction. Applying these structures, DICS will demonstrate more exceptional performance.

## Figures and Tables

**Table 1:** PSNR and SSIM comparisons for various approaches on the Set5 dataset at different sampling rates.

Approaches	Year	Rate = 1%		Rate = 4%		Rate = 10%		Rate = 25%		Avg.		SD	
		PSNR	SSIM	PSNR	SSIM	PSNR	SSIM	PSNR	SSIM	PSNR	SSIM	PSNR	SSIM
ReconNet	2016	18.09	0.4136	21.65	0.5455	24.68	0.6770	27.42	0.7812	22.95	0.6043	3.4743	0.1382
ISTA-Net+	2018	18.51	0.4427	23.51	0.6692	28.87	0.8437	34.69	0.9391	26.40	0.7237	6.0297	0.1889
SCSNet	2019	24.25	0.6469	28.98	0.8471	32.75	0.9081	36.77	0.9622	30.69	0.8411	4.6262	0.1193
CSNet*	2020	24.03	0.6380	28.78	0.8215	32.33	0.9016	36.55	0.9614	30.42	0.8306	4.6029	0.1218
OPINE-Net	2020	21.86	0.6010	28.06	0.8364	32.88	0.9263	37.47	0.9617	30.07	0.8314	5.7901	0.1406
ISTA-Net++	2021	20.90	0.5310	26.52	0.7909	31.30	0.8999	36.09	0.9554	28.70	0.7943	5.6339	0.1631
MR_CSGAN	2021	24.42	0.6451	28.86	0.8310	32.85	0.9157	37.59	0.9629	30.93	0.8387	4.8659	0.1213
AMP-Net	2021	23.11	0.6490	28.83	0.8376	33.40	0.9161	**38.01**	0.9585	30.84	0.8403	5.5171	0.1187
Ours		**24.57**	**0.6696**	**29.32**	**0.8539**	**33.70**	**0.9304**	37.95	**0.9658**	**31.38**	**0.8549**	4.9791	0.1144

The optimal and suboptimal results are emphasized in bold and underlined, respectively.

**Table 2:** PSNR and SSIM comparisons for various approaches on the Set14 dataset at different sampling rates.

Approaches	Year	Rate = 1%		Rate = 4%		Rate = 10%		Rate = 25%		Avg.		SD	
		PSNR	SSIM	PSNR	SSIM	PSNR	SSIM	PSNR	SSIM	PSNR	SSIM	PSNR	SSIM
ReconNet	2016	18.10	0.3911	20.72	0.4890	22.89	0.5971	25.35	0.7117	21.77	0.5472	2.6759	0.1197
ISTA-Net+	2018	18.31	0.4140	22.29	0.5851	26.36	0.7439	31.15	0.8807	24.53	0.6560	4.7665	0.1745
SCSNet	2019	22.84	0.5630	26.31	0.7226	29.25	0.8180	33.21	0.9105	27.90	0.7535	3.8128	0.1285
CSNet*	2020	22.71	0.5561	26.15	0.7138	28.94	0.8121	33.11	0.9009	27.73	0.7457	3.8113	0.1279
OPINE-Net	2020	21.47	0.5421	25.77	0.7276	29.18	0.8409	33.43	0.9251	27.46	0.7590	4.3970	0.1435
ISTA-Net++	2021	20.43	0.4736	24.62	0.6863	28.11	0.8131	32.37	0.9090	26.38	0.7205	4.3981	0.1630
MR_CSGAN	2021	23.07	0.5623	26.54	0.7243	29.40	0.8345	33.72	0.9261	28.18	0.7618	3.9045	0.1355
AMP-Net	2021	22.57	0.5733	26.61	0.7217	29.88	0.8129	**34.27**	0.9210	28.33	0.7572	4.2960	0.1275
Ours		**23.20**	**0.5793**	**26.82**	**0.7440**	**29.97**	**0.8522**	33.95	**0.9292**	**28.49**	**0.7762**	3.9615	0.1313

The optimal and suboptimal results are emphasized in bold and underlined, respectively.

**Table 3:** PSNR and SSIM comparisons for various approaches on the BSDS100 dataset at different sampling rates.

Approaches	Year	Rate = 1%		Rate = 4%		Rate = 10%		Rate = 25%		Avg.		SD	
		PSNR	SSIM	PSNR	SSIM	PSNR	SSIM	PSNR	SSIM	PSNR	SSIM	PSNR	SSIN
ReconNet	2016	19.18	0.4026	21.25	0.4905	23.11	0.5885	25.22	0.7031	22.19	0.5462	2.2344	0.1119
ISTA-Net+	2018	19.20	0.4054	22.22	0.5421	25.21	0.6899	30.01	0.8451	24.16	0.6206	3.9903	0.1641
SCSNet	2019	23.77	0.5481	26.49	0.6935	28.61	0.7841	31.94	0.9015	27.70	0.7318	2.9881	0.1292
CSNet*	2020	23.71	0.5431	26.11	0.6789	28.45	0.7779	31.69	0.8901	27.49	0.7225	2.9476	0.1277
OPINE-Net	2020	21.89	0.5000	25.00	0.6673	27.55	0.7903	31.20	0.8982	26.41	0.7140	3.4155	0.1481
ISTA-Net++	2021	21.08	0.4511	24.21	0.6340	26.85	0.7644	30.40	0.8813	25.64	0.6827	3.4264	0.1598
MR-CSGAN	2021	23.85	0.5443	26.35	0.6886	28.59	0.8018	32.28	0.9101	27.77	0.7362	3.0982	0.1357
Ours		**23.95**	**0.5527**	**26.52**	**0.7010**	**28.92**	**0.8145**	**32.48**	**0.9125**	**27.97**	**0.7452**	3.1427	0.1340

The optimal and suboptimal results are emphasized in bold and underlined, respectively.

**Table 4:** GPU running times of different methods for recovering a 256 × 256 image.

Methods	Avg.	SD	Platform
ReconNet	0.0195 s	-	Intel Xeon E5-1650 CPU + NVIDIA GTX980 GPU
CSNet	0.0751 s	-	AMD Core 3700X CPU + NVIDIA RTX3090 GPU
SCSNet	0.0927 s	-	
ISTA-Net+	0.0174 s	0.0091 s	Intel Xeon E5-2620 CPU + GeForce RTX1080Ti GPU
OPINE-Net	0.0350 s	0.0072 s	
ISTA-Net++	0.0410 s	0.0103 s	
MR-CSGAN	0.1210 s	0.0143 s	
Ours	0.0406 s	0.0095 s	

**Table 5:** PSNR comparisons of two structures on the Set14 dataset.

Methods	PSNR			
	Rate = 1%	Rate = 4%	Rate = 10%	Rate = 25%
w/o MSS	23.02	26.61	29.60	33.77
w/MSS	23.20	26.82	29.97	33.95

**Table 6:** GPU running times of two methods for recovering a 256 × 256 image.

Methods	Rate = 1%		Rate = 4%		Rate = 10%		Rate = 25%	
	Avg.	SD	Avg.	SD	Avg.	SD	Avg.	SD
LMSRB Based	0.0390 s	0.0094 s	0.0398 s	0.0095 s	0.0406 s	0.0095 s	0.0410 s	0.0097 s
MSRB Based	0.1189 s	0.0143 s	0.1200 s	0.0142 s	0.1210 s	0.0144 s	0.1219 s	0.0154 s

**Table 7:** Ablation study of different settings for the cost function. The experiments are conducted on the Set5 and Set14 datasets at a sampling rate of 10%.

Setting	Pixel Loss	Adv Loss	Perceptual Loss	PSNR	
				Set5	Set14
(a)	**✓**	**✕**	**✕**	33.47	29.80
(b)	**✓**	**✓**	**✕**	33.48	29.83
(c)	**✓**	**✕**	**✓**	33.65	29.96
(d)	**✓**	**✓**	**✓**	33.70	29.97

**Table 8:** Ablation study of different coefficient settings for the cost function. The experiments are conducted on the Set5 and Set14 datasets at a sampling rate of 10%.

Setting	*q*	*K*	*V*	PSNR	
				Set5	Set14
(e)	1	0.006	0.01	33.61	29.98
(f)	1	0.006	0.0001	33.67	29.95
(g)	1	0.06	0.001	32.64	29.39
(h)	1	0.0006	0.001	33.60	29.81
(i)	1	0.006	0.001	33.70	29.97

## Data Availability

https://doi.org/10.6084/m9.figshare.19874284 (accessed on 20 May 2022).
